# Supplementary material for: Identification and Functional Analysis of Two Chitin Synthase Genes in the Common Cutworm, Spodoptera litura
Source: Insects. 2020 Apr 17;11(4):253. doi: 10.3390/insects11040253 (PMC7240487; doi:10.3390/insects11040253)
Supplement: Supplementary file 1 [file insects-11-00253-s001.zip › Supplementry Figure/Figure S1.docx]

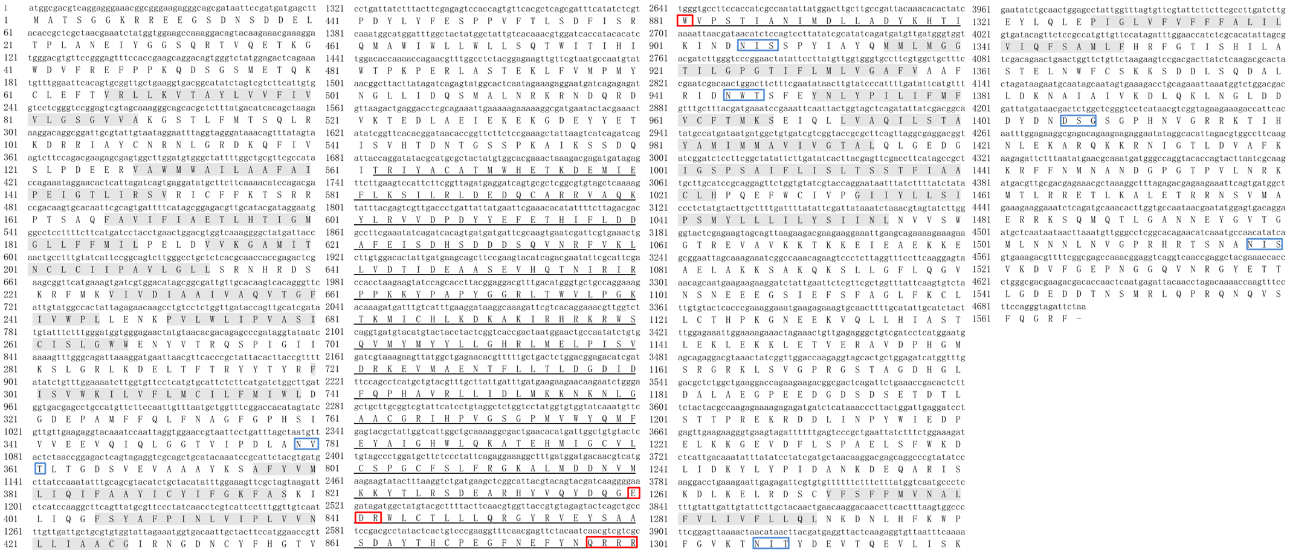


**Figure S1.** Nucleotide and deduced amino acid sequences of *SlCHS1* cDNA from *S. litura*. Numbers on the left side represent nucleotide and amino acid positions. The initiation codon (ATG) and termination codon (TAG) are indicated in black italics. The putative transmembrane helices predicted by TMHMM Server v. 2.0 are shaded grey. The six potential *N*-glycosylation sites are indicated by the blue box. The Chitin-synth_2 domain is indicated in a single line. The signature sequences (EDR and QRRRW) are indicated in the red box.
