# Supplementary material for: Identification and Functional Analysis of Two Chitin Synthase Genes in the Common Cutworm, Spodoptera litura
Source: Insects. 2020 Apr 17;11(4):253. doi: 10.3390/insects11040253 (PMC7240487; doi:10.3390/insects11040253)
Supplement: Supplementary file 1 [file insects-11-00253-s001.zip › Supplementry Figure/Table S1.docx]

Table S1. Sequences and relevant information used for phylogenetic analysis of the chitin synthase gene.

| **Genes** | **GenBank No** | ***Species*** |
| --- | --- | --- |
| *SlCHS1* | XP_022820392.1 | *Spodoptera litura* |
| *SlCHS2* | XP_022821184.1 | *Spodoptera litura* |
| *AgCHS1* | AFJ00066 | *Aphis Glycines* |
| *CmCHS1* | AJG44538 | *Cnaphalocrocis medinalis* |
| *CmCHS2* | AJG44539 | *Cnaphalocrocis medinalis* |
| *AaCHS1* | XP_001662200.1 | *Aedes aegypti* |
| *CqCHS1* | XP_001866798 | *Culex quinquefasciatus* |
| *DmCHS1* | NP_524233 | *Drosophila melanogaster* |
| *HaCHS1* | AKZ08594 | *Helicoverpa armigera* |
| *HaCHS2* | AKZ08595 | *Helicoverpa armigera* |
| *MbCHS1* | ABX56676 | *Mamestra brassicae* |
| *AmCHS1* | XP_395677.4 | *Apis mellifera* |
| *AmCHS2* | XP_001121152.2 | *Apis mellifera* |
| *PoCHS1* | AOE23678 | *Phthorimaea operculella* |
| *AqCHS1* | ABD74441 | *Anopheles quadrimaculatus* |
| *MsCHS1* | AAL38051 | *Manduca sexta* |
| *PxCHS1* | BAF47974.1 | *Plutella xylostella* |
| *SeCHS1* | AAZ03545 | *Spodoptera exigua* |
| *SeCHS2* | ABI96087 | *Spodoptera exigua* |
| *AaCHS2* | XP_001651163 | *Aedes aegypti* |
| *CqCHS2* | XP_001864594 | *Culex quinquefasciatus* |
| *DmCHS2* | NP_524209 | *Drosophila melanogaster* |
| *MsCHS2* | AAX20091 | *Manduca sexta* |
| *OfCHS1* | ACB13821 | *Ostrinia furnacalis* |
| *SfrCHS2* | AAS12599 | *Spodoptera frugiperda* |
| *TcCHS2* | AAQ55061 | *Tribolium castaneum* |
| *BdCHS2* | AGC38392 | *Bactrocera dorsalis* |
